# Electrically Tunable Nonequilibrium Optical Response of Graphene

**DOI:** 10.1021/acsnano.1c04937

**Published:** 2022-02-21

**Authors:** Eva A. A. Pogna, Andrea Tomadin, Osman Balci, Giancarlo Soavi, Ioannis Paradisanos, Michele Guizzardi, Paolo Pedrinazzi, Sandro Mignuzzi, Klaas-Jan Tielrooij, Marco Polini, Andrea C. Ferrari, Giulio Cerullo

**Affiliations:** † NEST, Istituto Nanoscienze-CNR and Scuola Normale Superiore, 56127 Pisa, Italy; ‡ Dipartimento di Fisica, Politecnico di Milano, P.zza Leonardo da Vinci 32, 20133 Milano, Italy; ¶ Dipartimento di Fisica, Università di Pisa, Largo Bruno Pontecorvo 3, 56127 Pisa, Italy; § Cambridge Graphene Centre, University of Cambridge, 9 JJ Thomson Avenue, Cambridge CB3 0FA, U.K.; ∥ Institute of Solid State Physics, Friedrich Schiller University Jena, Jena 07743, Germany; ⊥ L-NESS, Department of Physics, Politecnico di Milano, Via Anzani 42, Como 22100, Italy; # Catalan Institute of Nanoscience and Nanotechnology (ICN2), BIST & CSIC, Campus UAB, Bellaterra, Barcelona 08193, Spain; @ Istituto Italiano di Tecnologia, Graphene Laboratories, Via Morego 30, 16163 Genova, Italy; ∇ Istituto di Fotonica e Nanotecnologie, Consiglio Nazionale delle Ricerche, Piazza L. da Vinci 32, 20133 Milano, Italy

**Keywords:** graphene, cooling dynamics, hot electrons, tunable dynamics, optical phonons, phonon bottleneck

## Abstract

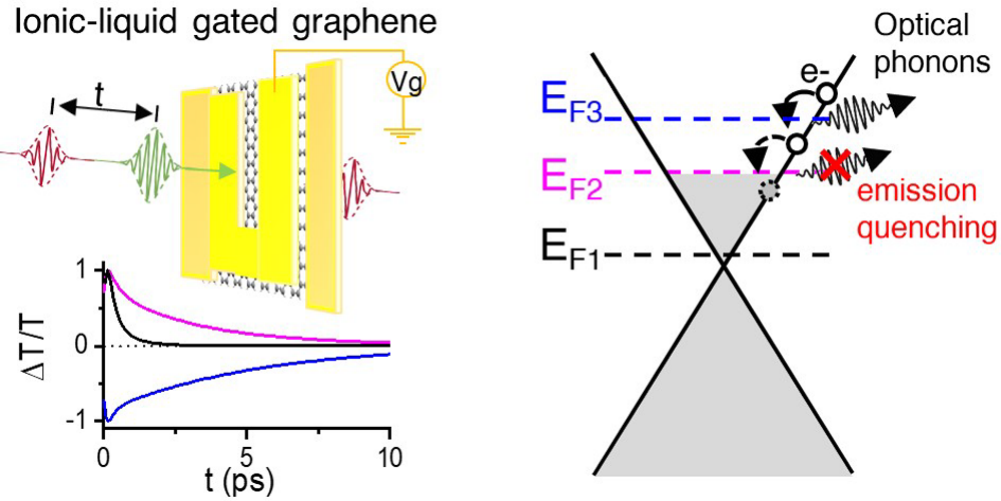

The ability to tune the optical response of a material via electrostatic gating is crucial for optoelectronic applications, such as electro-optic modulators, saturable absorbers, optical limiters, photodetectors, and transparent electrodes. The band structure of single layer graphene (SLG), with zero-gap, linearly dispersive conduction and valence bands, enables an easy control of the Fermi energy, *E*_F_, and of the threshold for interband optical absorption. Here, we report the tunability of the SLG nonequilibrium optical response in the near-infrared (1000–1700 nm/0.729–1.240 eV), exploring a range of *E*_F_ from −650 to 250 meV by ionic liquid gating. As *E*_F_ increases from the Dirac point to the threshold for Pauli blocking of interband absorption, we observe a slow-down of the photobleaching relaxation dynamics, which we attribute to the quenching of optical phonon emission from photoexcited charge carriers. For *E*_F_ exceeding the Pauli blocking threshold, photobleaching eventually turns into photoinduced absorption, because the hot electrons’ excitation increases the SLG absorption. The ability to control both recovery time and sign of the nonequilibrium optical response by electrostatic gating makes SLG ideal for tunable saturable absorbers with controlled dynamics.

## Introduction

Single-layer graphene (SLG) has unique optoelectronic properties,^1−3^ which stem from the physics of its massless Dirac fermions. These include high electron mobility (>100 000 cm^2^ V^–1^ s^–1^ at room temperature (RT)^4−7^), broadband optical absorption,^8^ tunability of Fermi energy, *E*_F_, via electrostatic gating,^9^ resulting from the linear dispersion of conduction (CB) and valence bands (VB), and a vanishing density of states at the Dirac point.^10^

Light absorption in SLG is due to the interplay of intraband^11−13^ and interband^14,15^ transitions. In undoped SLG, the first ones dominate in THz^16^ and microwaves,^17^ the second^14^ in near-infrared (NIR)^15^ and visible (VIS).^14^ Electrical control of *E*_F_, by exploiting the band-filling effect,^18^ allows one to vary the density of electronic states available for both intraband^15^ and interband transitions,^3,18^ thus affecting the linear absorption of SLG over a broad range from THz^19−23^ to NIR^24−27^ and VIS.^28^ This led to the development of SLG-based electro-optic modulators,^3,24,25,27,29−33^ which can reach higher modulation speed (up to 200 GHz^34^) than LiNbO_3_^35^ and Si^36^ because of the superior mobility of SLG charge carriers, with high modulation depths both in amplitude (up to ∼60*%*)^3,19,21,24,26^ and phase (∼65°).^3,31^

SLG also exhibits large nonlinear optical response^37−41^ because of a strong coupling to light. The third-order nonlinear optical susceptibility of SLG in the NIR at 0.7 eV is^37^ χ^3^ ∼ 5 × 10^–18^ m^2^V^–2^, several orders of magnitude higher than in dielectrics (e.g., ∼ 10^–22^m^2^V^–2^ for SiO_2_^42^) and atomically thin semiconductors (e.g., ∼ 6 × 10^–20^ m^2^ V^–2^ for single-layer WSe_2_^43^). Nonlinearities of higher order have been exploited for high-harmonics generation in SLG.^38,39^ The strong nonlinear response results also in saturable absorption,^44^ optical Kerr effect,^45^ and optical bistability,^46,47^ i.e., the ability to provide two stable optical outputs for a specific light input.^48^*E*_F_ control via external gating allows one to tune the nonlinear optical response of SLG, resulting in gate-tunable third-harmonic generation^37,40,41^ and four-wave-mixing.^49^

The *E*_F_ dependence of the transient absorption properties of SLG when brought out of equilibrium remains still largely unexplored, with studies limited to the THz range,^50−53^ discussing the tuning of intraband photoconductivity with *E*_F_.^50−52^ The modulation of interband absorption in NIR and VIS is more challenging to study because *E*_F_ needs to be ∼0.5 eV in order to cross the Pauli blocking threshold, above which the nonequilibrium optical properties have been only theoretically explored.^54^

The nonequilibrium optical response of SLG is crucial for optoelectronic applications, such as photodetectors,^55^ relying on the relaxation dynamics of photoexcited charge carriers. Numerous ultrafast optical spectroscopy experiments were performed on SLG^56−60^ to investigate the charge-carriers relaxation dynamics by looking at the modifications it induces on SLG absorption. In a pump–probe experiment, the system is photoexcited by an optical pulse, the pump, whose duration is to be shorter than the time scale of the relaxation processes under investigation. The relaxation of the photoexcited system is then monitored by detecting the absorption of a second optical pulse, the probe, as a function of the time delay with respect to the pump pulse.^61^

In SLG, interband absorption of the pump pulse induces out-of-equilibrium distributions of holes (h) and electrons (e) in VB and CB, respectively, peaked at ± *ℏω*_pump_/2, where *ℏω*_pump_ is the pump photon energy. Carrier-carrier scattering drives the ultrafast e–h thermalization on a time-scale τ_th_ < 20 fs^60^ from out of equilibrium, to hot Fermi–Dirac distributions (HFD) with defined electronic temperature, *T*_e_. The HFD can be detected in a pump–probe experiment as a photobleaching (PB) signal,^56−58^ i.e., decreased probe absorption compared with equilibrium, because of Pauli blocking of interband transitions caused by the photogenerated e/h. The excess energy of the hot charge-carriers is released to the lattice via electron–phonon scattering with optical phonons,^62−64^ anharmonically coupled to acoustic phonons.^62−65^ Hot carriers’ cooling occurs on a few-ps time-scale^56−58,60,65^ and is influenced, through the activation of additional relaxation channels, by the dielectric environment (e.g., via near-field coupling to hyperbolic optical phonons of substrate or encapsulant material^66^). Defects can also accelerate the cooling via electron–phonon interaction, by acting as scattering centers mediating the direct coupling of the hot charge carriers with finite momentum acoustic phonons.^67−69^ This process, referred to as supercollision,^67−69^ accelerates the cooling for increasing defect density.^70^

Here we investigate the *E*_F_ dependence of the nonequilibrium optical response of SLG in the NIR range between 0.729 and 1.240 eV (1000–1700 nm), exploiting ionic liquid gating to tune *E*_F_ from −650 to 250 meV, thus exceeding the Pauli blocking threshold for interband absorption, achieved when |*E*_F_| = *ℏω*_probe_/2, where *ℏω*_probe_ is the energy of the probe beam. Applying ultrafast pump–probe spectroscopy with 100 fs time resolution, we detect the changes with *E*_F_ of amplitude and sign of the differential transmission (Δ*T*/*T*), as well as of its relaxation dynamics. Starting from not intentionally doped SLG and increasing *E*_F_, we first observe a rise in PB amplitude (Δ*T*/*T* > 0) with a slow-down of its relaxation dynamics. Above the Pauli blocking threshold, photoexcitation has an opposite effect on SLG, activating additional absorption channels, as shown by the appearance of photoinduced absorption (PA) (Δ*T*/*T* < 0). The Δ*T*/*T* changes are assigned to the *E*_F_ dependence of the hot carriers cooling dynamics, simulated considering relaxation through emission of optical phonons. The gate tunability of the nonequilibrium optical response is key for optoelectronic applications, such as saturable absorbers (SA) with gate-tunable response.

## Results and Discussion

**Figure 1:**
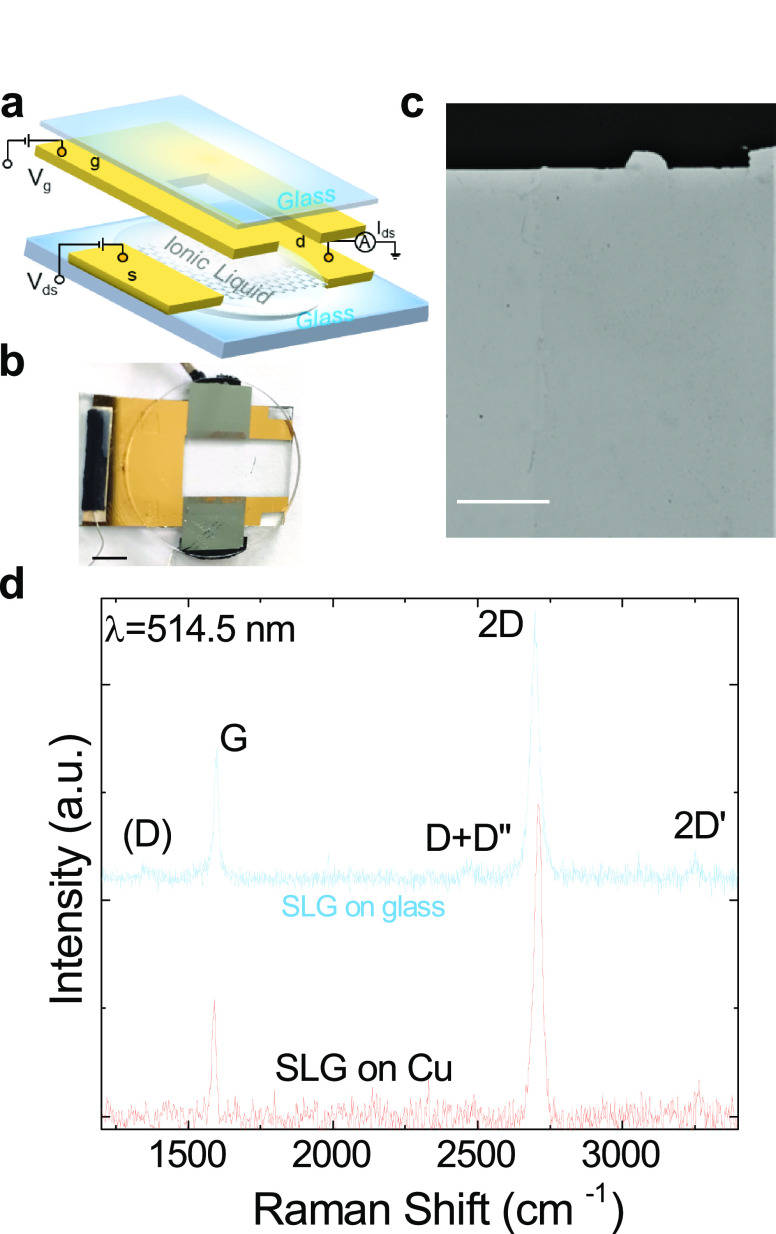
(a) Schematic of device with source (s), drain (d) and gate (g) contacts used to tune the SLG *E*_F_ while measuring its transmission properties. (b) Photo of representative device. Scale bar 5 mm. (c) Image of transferred SLG in a region near the drain contact (dark area on the top). Scale bar: 100 μm. (d) 514.5 nm Raman spectrum of SLG as-grown and transferred on glass.

We modulate *E*_F_ by means of the electrostatic field effect^71^ using an ionic-liquid top-gated field effect transistor (FET) sketched in Figure 1a. The top-gate geometry, with diethylmethyl (2-methoxyethyl) ammoniumbis(trifluoromethylsulfonyl)imide (C_6_H_20_F_6_N_2_O*_5_*S**_2_) as ionic liquid, is chosen to allow light measurements in transmission through a ∼1 cm^2^ optical window. Large area (8 mm × 8 mm) SLG is prepared by chemical vapor deposition (CVD) as for ref (72). The device fabrication follows ref (73). Figure 1b is a photo of the device, and Figure 1c an optical image of the transferred SLG, showing no macroscopic tearing nor folding.

Both as-grown and transferred SLG are characterized with a Renishaw InVia Raman spectrometer using a 50× objective, a CW laser at 514.5 nm, with power on the sample <0.5 mW to exclude heating effects. The Raman peaks are fitted with Lorentzians, with error bars derived from the standard deviation across 6 measurements and the spectrometer resolution ∼1 cm^–1^. The Raman spectrum of as-grown SLG on Cu is in Figure 1d, after Cu photoluminescence removal.^74^ The 2D peak is a single Lorentzian with full-width half-maximum FWHM(2D) ∼31 ± 3 cm^–1^, a signature of SLG.^75^ The G peak position Pos(G) is ∼1586 ± 2 cm^–1^, with FWHM(G) ∼ 16 ± 3 cm^–1^. The 2D peak position, Pos(2D), is ∼2704 ± 4 cm^–1^, while the 2D to G peak intensity and area ratios, *I*(2D)/*I*(G) and A(2D)/A(G), are 3.1 ± 0.4 and 6.2 ± 0.7. No D peak is observed, indicating negligible Raman active defects.^76,77^

The Raman spectrum of SLG transferred on glass is in Figure 1d. The 2D peak retains its single-Lorentzian line shape with FWHM(2D) ∼ 36 ± 1 cm^–1^. Pos(G) ∼ 1597 ± 1 cm^–1^, FWHM(G) ∼ 15 ± 1 cm^–1^, Pos(2D) ∼ 2696 ± 1 cm^–1^, *I*(2D)/*I*(G) ∼ 2 ± 0.2 and *A*(2D)/*A*(G) ∼ 4.9 ± 0.3, indicating *p-*doping with *E*_F_ ∼ −230 ± 80 meV.^71,78^*I*(D)/*I*(G) ∼ 0.06 ± 0.05 corresponds^79^ to a defect density ∼2.6 ± 1.9 × 10^10^ cm^–2^ for excitation energy 2.41 eV and *E*_F_ = −230 ± 80 meV. Pos(G) and Pos(2D) are affected by the presence of strain.^80^ For uniaxial(biaxial) strain, Pos(G) shifts by ΔPos(G)/Δε ∼ 23(60)cm^–1^%^–1^.^80,81^ Pos(G) also depends on *E*_F_.^9,71^ The average doping as derived from *A*(2D)/*A*(G), FWHM(G) and *I*(2D)/*I*(G), should correspond to Pos(G) ∼ 1588 ± 1 cm^–1^ for unstrained graphene.^9,71^ However, we have Pos(G) ∼ 1597 ± 1 cm^–1^, which implies a contribution from uniaxial (biaxial) strain ∼0.16 ± 0.02% (0.4 ± 0.04%).^80,81^

**Figure 2:**
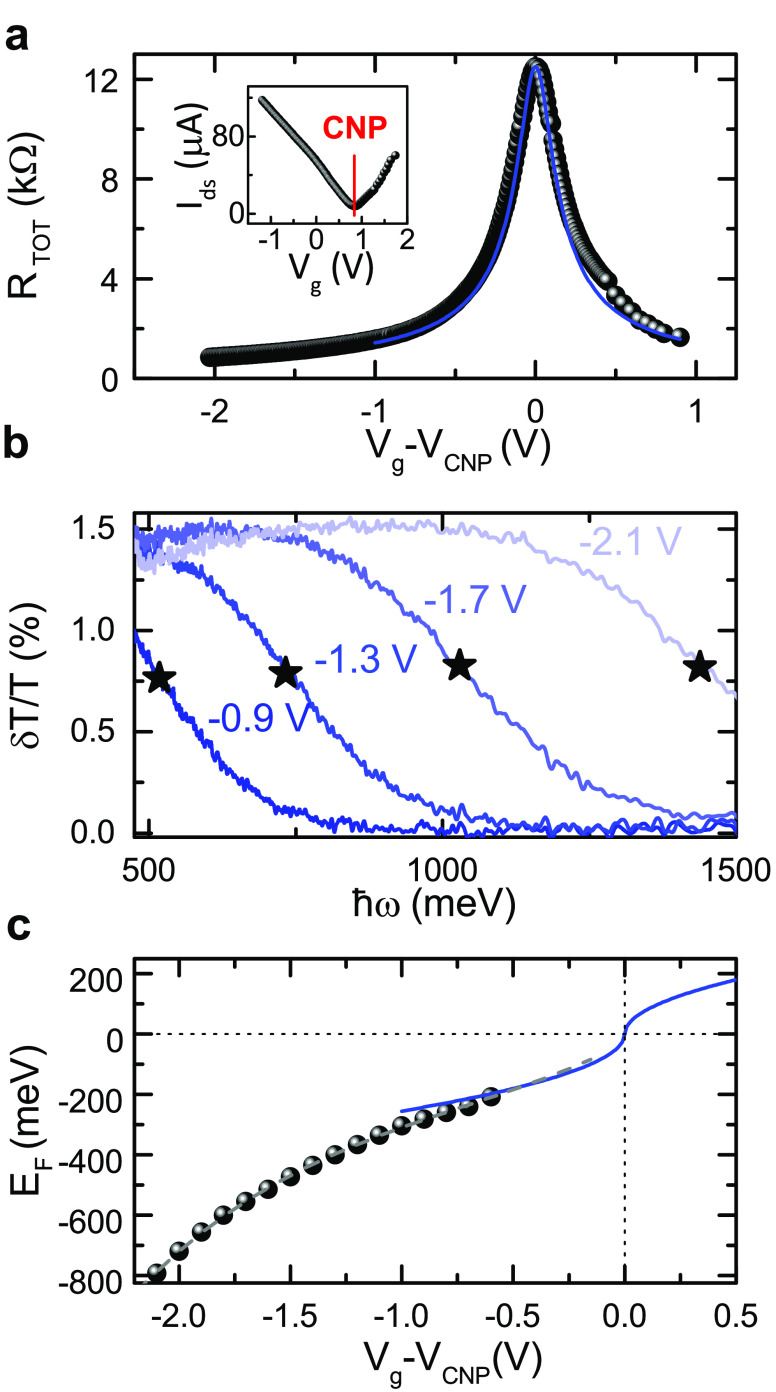
(a) Total resistance *R*_TOT_ as a function of *V*_g_-*V*_CNP_ (black dots) with Drude model fitting (blue solid line) to estimate residual charge carrier density, *n*_0_, and gate capacitance *C*. Inset, *I*_ds_ for *V*_ds_ = 100 mV. (b) *δT*/*T* for different *V*_*g*_–*V*_CNP_ (indicated next to the curves) as a function of photon energy *ℏω*, showing the gate tunability of the absorption edge for interband transitions; (c) *E*_F_ determined from *δT*/*T* as a function of *V*_*g*_–*V*_CNP_ (full dots) interpolated by *f*(*x*) = *A*_0_ + *A*_1_x + *A*_2_x^2^ + *A*_3_x^3^ with *A*_0_ = −0.03 eV, *A*_1_ = 0.37 eV/V *A*_2_ = 0.17 eV/V^2^*A*_3_ = 0.08 eV/V^3^ (gray dashed line), together with the trend (blue solid line)  where *n*_*e*_(*V*_g_) = (*V*_g_ – *V*_CNP_)(*C*/*e*) is the gate-tunable charge carrier density

The gate voltage, *V*_g_, polarizes the ionic liquid leading to the formation of electrical double layers (EDLs), near SLG and Au interfaces,^71,82^ that modulate the carrier density. Since the EDL thickness is ∼1 nm for ionic liquids,^83,84^ the solid–liquid interfacial electric field and the induced charge densities on the surface reach values as large as^22,83^ ∼10–20 MV cm^–1^ and 10^14^ cm^–2^ even at moderate *V*_g_ ∼ 1–2 V. The transfer characteristics of our device for source-drain bias *V*_ds_ = 100 mV is in Figure 2a. This exhibits a typical ambipolar behavior, as seen by the *V*-shaped gate dependence of the source-drain current *I*_ds_. The channel resistance peaks at *V*_CNP_ = 0.84 V, corresponding to the charge neutrality point (CNP), where the density of states in SLG reaches its minimum.^22,23,85^*V*_CNP_ depends on *E*_F_, on the gate-metal work function,^22^ and on the choice of contact materials.^86^

In order to determine *E*_F_ as a function of *V*_g_, we measure the static transmission *T* in NIR (500–1500 meV) with an Agilent Cary 7000 spectrometer. Figure 2b plots a selection of transmission spectra for different *V*_*g*_, compared with that at the CNP, evaluated as . *T* increases with respect to the CNP, i.e., *δT*/*T* > 0, when absorption is inhibited by Pauli blocking, due to e in CB (*n-*doping) or h in VB (*p-*doping). In terms of probe photon energy, this corresponds to *ℏω*_probe_ < 2|*E*_F_|. We estimate *E*_F_ considering that *δT*/*T* halves^73^ for *ℏω*_probe_ = 2|*E*_F_| at values indicated by black stars in Figure 2b. For probe photon energies *ℏω*_probe_ < 2|*E*_F_|, interband absorption is blocked, and the sample has *T* ∼ 99.6–99.8*%*, with ∼0.2–0.4*%* residual absorption being attributed to intraband transitions enabled by disorder.^73^ The *T* modulation due to the bleaching of interband absorption is ∼1.5*%* against the ∼2.3*%* expected for suspended SLG,^8^ because of the presence of the glass substrate and of diethylmethyl (2-methoxyethyl) ammoniumbis(trifluoromethylsulfonyl)imide,^87^ with a refractive index ∼1.418–1.420.^88^

*E*_F_ extracted from the *T* measurements is plotted in Figure 2c as a function of *V*_g_. At *V*_g_ = 0 V, there is a *p-*doping *E*_F_ ∼ −250 meV in agreement with the Raman estimation (∼−230 ± 80 meV) without ionic liquid. From the analysis of the charge-transfer curve with the Drude model as in ref (23) (see blue line in Figure 2a), we evaluate a residual *n*_0_ = 5.6 × 10^11^cm^–2^, responsible for the finite conductivity at the CNP, and a gate capacitance *C* = 766 nF cm^–2^. We note this is a typical *n*_0_ for as-grown and transferred SLG.^89^ A lower residual doping ∼10^11^ cm^–2^ can be achieved with cleaning techniques,^6^ not used here. The finite electrical conductivity and doping at the CNP^22,23^ are due to electron–hole puddles,^90^ caused by charged impurities^91^ located either in the dielectric, or at the SLG/dielectric interface.^91^ Near the CNP, for |*V*_g_–*V*_CNP_| < 0.6 V, the interband absorption edge is outside the spectral window of our *δT*/*T* measurements, and we evaluate^8^, with *v*_F_ the Fermi velocity, directly from the gate tunable charge carrier density *n*_*e*_(*V*_g_) = (*V*_g_ – *V*_CNP_)(*C*/*e*), with *e* the electron charge, see blue solid line in Figure 2c. At high gate voltages |*V*_g_ – *V*_CNP_| > 0.9 V, the disagreement between calculated *E*_F_ and the values obtained from *δT*/*T* is attributed to the dependence of mobility on charge carrier density,^6^ not included in the analysis of the transport properties used for the calculated *E*_F_, that is valid near the CNP and fails to describe the sample behavior for |*V*_g_ – *V*_CNP_| > 0.9 V. Accordingly, we assume the values extracted from *δT*/*T* (Figure 2c gray dashed line) in the range |*V*_g_ – *V*_CNP_| > 0.6 V, and those calculated from *n*_*e*_(*V*_g_) (Figure 2c, blue solid line), in the range |*V*_g_ – *V*_CNP_| < 0.6 V. At each *V*_g_ of Figure 2. We also monitor the source-drain current *I*_ds_ (inset of Figure 2a) to determine the empirical relation with *E*_F_. The transfer curve that we obtain allows us to track *E*_F_ by monitoring *I*_ds_ during all subsequent measurements. We test the *E*_F_ tunability of the ionic liquid top-gate device up to −800 meV, corresponding to a wide range of charge carrier densities from ∼4.5 × 10^12^ cm^–2^ (*n-*doping) to ∼−4.7 × 10^13^ cm^–2^ (*p-*doping), much wider than possible with a 285 nm SiO_2_ back gate, usually limited to ±6 × 10^12^cm^–2^ by the gate capacitance.^85^ We got similar *E*_F_(*V*_*g*_) in ref (73) from Raman spectra and NIR transmission.

**Figure 3:**
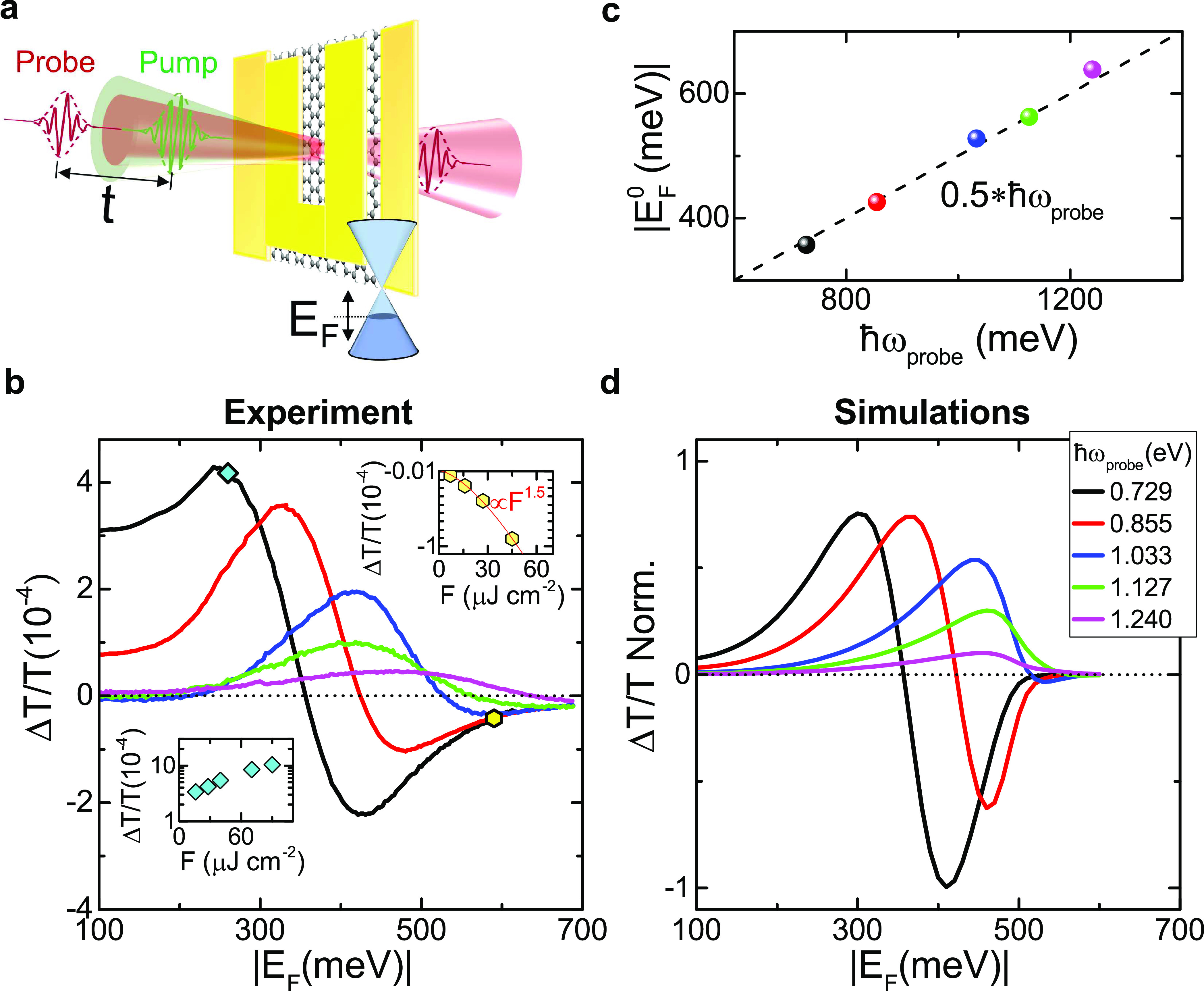
(a) Sketch of pump–probe experiment on SLG with tunable *E*_F_ controlled by V_g_. (b) Experimental Δ*T*/*T* at *t* = 150 fs as a function of |*E*_F_| for *p-*doping acquired at different *ℏω*_probe_ = 0.729, 0.855, 1.033, 1.127, 1.240 eV (legend of panel d) for a pump fluence *F* ∼ 28 μJ cm^–2^. Top-right inset: fluence dependence of Δ*T*/*T* amplitude above Pauli blocking for pump absorption, at *ℏω*_probe_ = 0.729 eV and *E*_F_ = −590 meV (hexagonal yellow symbol in main figure), together with a superlinear power-law dependence on *F* (solid red line). Bottom-left inset: fluence dependence of Δ*T*/*T* amplitude, for *ℏω*_probe_ = 0.729 eV and *E*_F_ = −260 meV (cyan rhombus symbol in main figure). (c) |*E*_F_^0^| at Δ*T*/*T* = 0, extracted from panel b, as a function of *ℏω*_probe_. (d) Simulated Δ*T*/*T* at *t* = 150 fs as a function of |*E*_F_| for *p-*doping at the same *ℏω*_probe_ as in panel b.

We perform ultrafast pump–probe spectroscopy as sketched in Figure 3a. The pump is a 100 fs NIR pulse centered at *ℏω*_*pump*_ = 0.8 eV, while the probe spectrum covers *ℏω*_probe_ = 0.729–1.240 eV (see Methods for details). The relaxation dynamics is monitored through the differential transmission  evaluated from the probe transmission with (*T*_pump–ON_) and without (*T*_pump–OFF_) pump excitation, after a time delay *t* between probe and pump pulses, varied with an optical delay line. Given that the pulses duration exceeds the time-scale of carrier–carrier thermalization,^60^ we can assume charge carriers thermalized to HFDs and investigate their cooling dynamics.

Figure 3b plots Δ*T*/*T* at *t* = 150 fs, chosen as the delay at which the maximum signal amplitude is reached for *V*_g_ = 0 V. The signal is plotted as a function of *E*_F_ for different probe photon energies. Since the transient response is symmetric with respect to the CNP for *n-* and *p-* doping and our SLG is *p-*doped at *V*_g_ = 0, we explore negative *E*_F_ in order to reach higher |*E*_F_| by applying a smaller *V*_g_. We observe a strong modulation of Δ*T*/*T* with *E*_F_, higher at the low-energy tail of the probe pulse, with the signal changing from 4 to −2 × 10^–4^ (see the curve at 0.729 eV in Figure 3b). The signal amplitude decreases for increasing *ℏω*_probe_, as expected for a thermal distribution of carriers.^58^ In all the probed range, near the CNP, we observe, as expected, a PB signal, i.e., Δ*T*/*T*(*t*) > 0. By increasing |*E*_F_|, first PB increases in amplitude, and then a change of sign occurs at a threshold |*E*_F_| dependent on the probe photon energy. The Fermi energy at which the sign change occurs, |*E*_F_^0^| in Figure 3c, corresponds to *ℏω*_probe_/2, i.e., the Pauli blocking threshold for probe photons. Above this, the pump pulse, exciting e (h) to higher (lower) energy states, partially unblocks the probe interband absorption, otherwise inhibited, resulting in a PA signal, i.e., Δ*T*/*T* < 0. The PA intensity increases with *E*_F_ up to a peak, whose position in terms of *E*_F_ raises with probe photon energy. A constant Δ*T*/*T* ∼ −1 × 10^–5^ is then approached in the high |*E*_F_| limit (*E*_F_ < −690 meV) in all the probed range.

We note that ref (93) reported a study of *E*_F_ dependence of the transient optical properties of SLG, without reaching Pauli blocking, i.e., |*E*_F_| > *ℏω*_probe_/2, which we investigate here, revealing the PA regime. Our results show that, by varying *V*_g_, we can not only control the relaxation dynamics of SLG, but also change the Δ*T*/*T* sign.

Above the Pauli blocking threshold for pump interband transitions (|*E*_F_| ≥ 400 meV for *ℏω*_pump_ = 800 meV), Δ*T*/*T* is expected to vanish, because the pump should not be able to photoexcite SLG. However, a finite value is observed, caused by residual pump absorption, related to both extrinsic^14,73,92^ and intrinsic^14,94^ effects. Among the former, charged impurities and scatterers (e.g., edge defects, cracks, vacancies) can induce residual conductivity^73,92^ activating intraband absorption. Amongst the latter is the residual absorption from the tail of the carrier Fermi distribution, i.e., off-resonance absorption, which has a finite broadening at RT.^94^ The fluence dependence of Δ*T*/*T* at *ℏω*_probe_ = 0.729 eV in the inset of Figure 3b is superlinear above the threshold for Pauli blocking of pump absorption (as measured at |*E*_F_| = 590 meV), suggesting a non-negligible contribution from two-photon absorption.^95^ This could also explain the vanishing signal when approaching |*E*_F_| = 800 meV (the Pauli blocking threshold for two-photon absorption).

While height and width of PB and PA bands slightly change with *ℏω*_probe_, we observe similar features in all the probed range upon increasing |*E*_F_|: an increase of PB, followed by a decrease, and a sign change above the Pauli blocking threshold for probe absorption. The measurements are performed using a low excitation fluence (28 μJ cm^–2^) to work in a perturbative regime corresponding to *T*_e_ < 1000K, thus reducing the impact of the hot-phonon bottleneck and focusing on the electron-optical phonon cooling. The amplitude of Δ*T*/*T* increases by increasing the excitation fluence, as for the inset of Figure 3b, by almost 2 orders of magnitude for the PA signal (see top-right inset of Figure 3b at |*E*_F_| = 590 meV) and 1 order of magnitude for the PB signal (see bottom-left inset of Figure 3b at |*E*_F_| = 260 meV). We previously reported the use of ungated SLG as SA (GSA) in mode-locked lasers, in which an absorption modulation ≤1.3% is sufficient to induce and control the pulsed (mode-locked) regime.^44^ The ability to electrically control amplitude, sign, and recovery time of Δ*T*/*T* in a GSA is thus of practical relevance for optimizing mode-locking for stability, pulse width, and average output power.

To understand the *E*_F_ dependence of the nonequilibrium optical response of SLG, we calculate Δ*T*/*T* (see Methods for details) as a function of initial carrier density *n*_*e*_, related^8^ to *E*_F_ by . Δ*T*/*T* in Figure 3d is computed from the changes in optical conductivity, Δσ, induced by photoexcitation as a function of *E*_F_. To evaluate Δ*T*/*T* at *t* = 150 fs we consider the charge carriers as distributed in energy and momentum along a HFD with a time-dependent chemical potential, μ_*c* _, and *T*_*e*_(*t*) > RT. Our model takes into account that, even though the pump fluence is constant, the initial *T*_e_ changes with *E*_F_ due to the change of pump absorption. We consider the absorption from the tail of the Fermi–Dirac distribution as source of residual pump absorption for |*E*_F_| > 400 meV. The charge carrier distribution modification with *E*_F_ is sufficient to reproduce qualitatively the experimental PB signal increase, the change of sign at *ℏω*_probe_/2, and the PA decrease for *E*_F_ > 400 meV, Figure 3d.

Our data and model indicate that the PA signal amplitude is maximized at *E*_F_ ∼ 0.4*ℏω*_probe_, unlike claimed in ref (93), i.e., that the maximum occurs at *E*_F_ = 0.5*ℏω*_pump_. We attribute this difference to the coarser sampling of *E*_F_ in ref (93) (50 meV steps against our 4 meV, Figure 3b), to the fact that ref (93) used a reflection geometry, mixing contributions from transient reflection and transmission with opposite sign,^96^ and to the larger probe photon energy at which intraband transitions contribute with opposite sign to that of interband transitions.^97^ Furthermore, the *E*_F_ thresholds that control the Δ*T*/*T* amplitude and sign are identified by *ℏω*_probe_, not by *ℏω*_pump_ as incorrectly stated in ref (93), since *ℏω*_pump_ has no role in defining the cooling dynamics of the HFD after the first ultrafast (<100 fs) step of electron–electron thermalization.^60,63^

**Figure 4:**
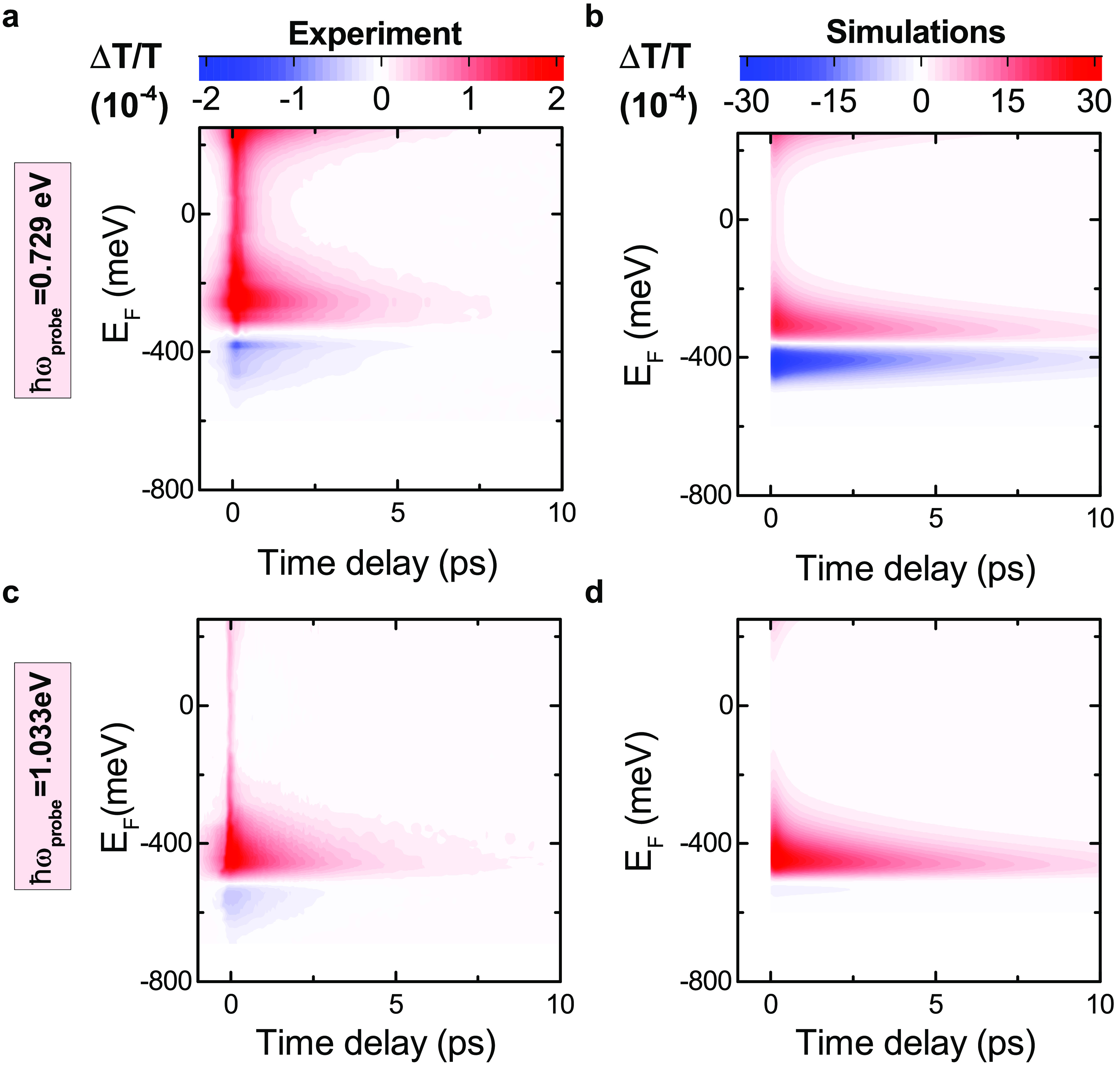
Time-evolution of Δ*T*/*T*(*t*) for different *E*_F_ at (a,b) *ℏω*_probe_ = 0.729 eV, with (a) experiment, and (b) simulations, and (c,d) *ℏω*_probe_ = 1.033 eV, with (c) experiment and (d) simulations. *t* = 0 corresponds to the pump arrival. The signal at negative delays indicates a finite build-up time, exceeding the pump–probe time duration at |*E*_F_| = 250 meV for *ℏω*_probe_ = 0.729 eV and 350 meV for *ℏω*_probe_ = 1.033 eV, approaching the Pauli blocking *E*_F_.

To examine the dependence of the cooling dynamics on *E*_F_, we monitor Δ*T*/*T* as a function of pump–probe delay. Figures 4a,c show the gate-dependent relaxation dynamics at *ℏω*_probe_ = 0.729, 1.033 eV, lower and higher than the pump photon energy. At both energies, the relaxation dynamics progressively slows down with increasing |*E*_F_|, evolving from a biexponential to a monoexponential decay, due to a reduction of the fast decay component. We can appreciate this slowdown by noting that, to see a signal reduction by a factor 10, we need to wait ∼1 ps at |*E*_F_| = 100 meV and ∼5 ps at 300 meV. Both signal intensity and relaxation dynamics are symmetric for *n-* or *p-*doping, as a consequence of the CB, VB symmetry.

The observed gate-dependence can be qualitatively explained considering that, for increasing |*E*_F_|, the excess energy of the photoexcited charge carriers with respect to equilibrium is reduced, affecting the scattering with optical phonons that drives the cooling. To gain a deeper insight into the phenomena responsible for quenching the fast relaxation component, we solve a set of phenomenological equations of motion (EOMs)^98^ for *T*_*e*_ and for the occupation of the phonon modes. We include the optical phonon modes at the K and Γ points of the SLG Brillouin zone, and we consider that they can be emitted/absorbed by e and h and decay into acoustic modes due to anharmonic coupling^62,63,65^ (see Methods).

We calculate the time-evolution of the differential conductivity for several values of μ_*c* _, corresponding to *E*_F_, (i.e., μ_*c*_ at T_*e*_ = 0^94^), in the range 250 to −650 meV. The results in Figure 4b,d explain the observed slowdown of the dynamics with increasing *E*_F_, with the saturation of the phase space for optical phonon-emitting electronic transitions. As *E*_F_ increases, there are fewer carriers with an energy high enough (>160 meV) to emit an optical phonon, and optical phonon emission is quenched. This is a fundamental process, not dependent on the SLG substrate, like supercollision cooling through defects,^99^ nor on its dielectric environment, like the cooling to hyperbolic phonons in hBN-encapsulated SLG.^66^ It is determined by the intrinsic coupling of e with the K and Γ phonons.^100^ The initial increase of PB amplitude with *E*_F_ in Figure 3b,c is a consequence of the quenching of relaxation via optical phonons,^60,63^ which reduces the initial fast decay.

Figure 4 also shows that |*E*_F_^0^| is independent of *t*, both in experiments and simulations. The vanishing Δ*T*/*T* does not correspond to zero absorption, but it means that the conductivity remains at its equilibrium value for all delays. For *t* > 0, the e system is photoexcited. This can happen only because the e distribution undergoes a time-evolution such that the conductivity remains time-independent at *ℏω* = 2|*E*_F_|.

**Figure 5:**
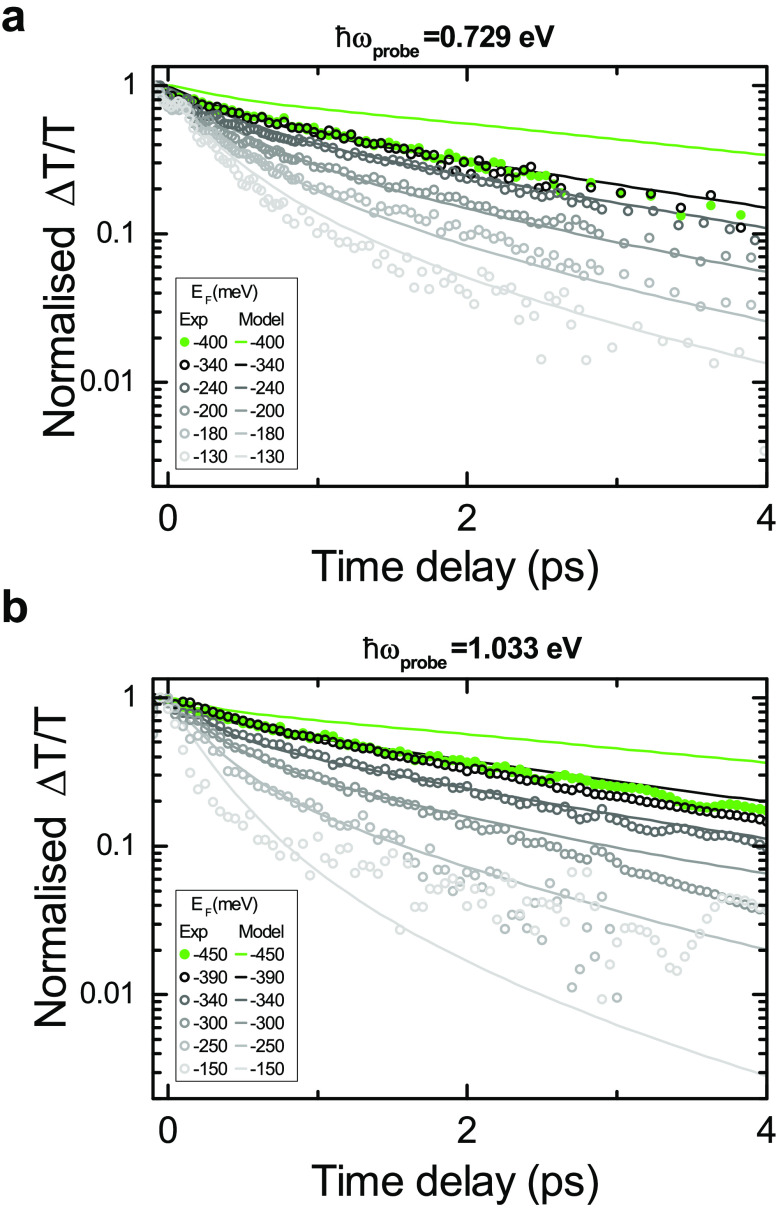
(a,b) Experimental (colored dots) and theoretical (solid lines) Δ*T*/*T* at different *E*_F_ for (a) *ℏω*_probe_ = 0.729 eV and (b) *ℏω*_probe_ = 1.033 eV for pump–probe time delays between −500 fs and 5 ps (pump arrival at *t* = 0).

Figure 5a shows that for *E*_F_ > 340 meV and *ℏω*_probe_ = 0.729 eV, the simulations predict a further slowdown of the relaxation dynamics, not observed in our experiments. These all saturate to a similar decay trend independent of *E*_F_ (see overlapping black and green dots in Figure 5). Analogous behavior is found at all *ℏω*_probe_, provided that when we increase *ℏω*_probe_, we tune *E*_F_ to higher levels to find overlapping decay dynamics (see Figure 5b). To understand the saturation of this slowdown, we need to consider that additional relaxation channels may start playing a role once the cooling via optical phonons gets slower. Defects can accelerate cooling,^67−70^ mediating the scattering with acoustic phonons of finite momentum and energy.^69^ This supercollision mechanism^67−70^ may become the dominating process once optical phonon emission is quenched. Cooling times ∼4 ps are expected for supercollision cooling^67^ in SLG with *E*_F_ ∼ 400 meV and mobility of a few thousand cm^2^ V^–1^ s^–1^, as that of our device. The *E*_F_ independence of the decay dynamics in the high *E*_F_ limit could be explained by the lack of dependence on carrier density of the supercollision cooling time away from the Dirac point.^67^ According to refs (78) and (79), the e scattering time with defects in SLG is not expected to significantly change with *E*_F_.

**Figure 6:**
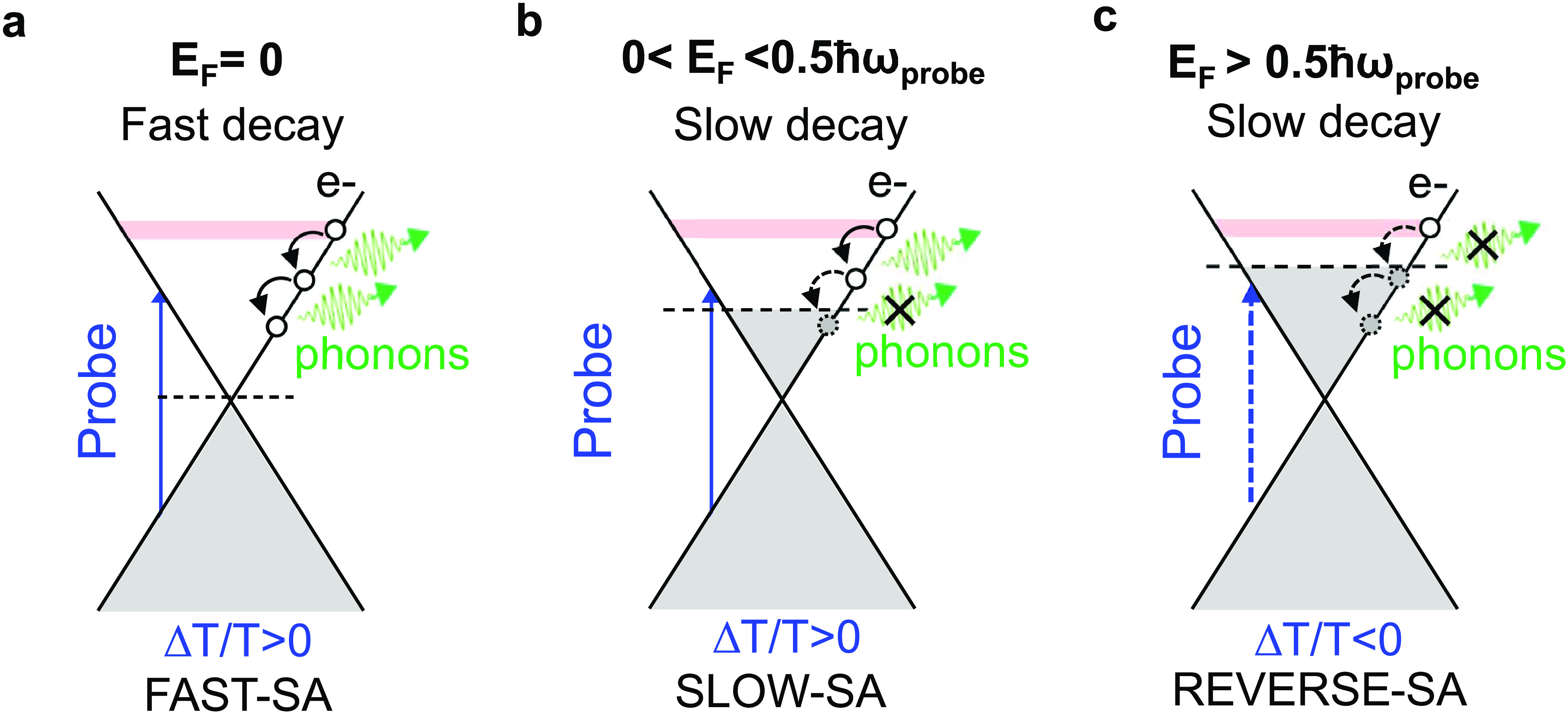
(a–c) Sketch of interband absorption of a NIR probe pulse (vertical blue arrow) within the SLG Dirac cones populated at equilibrium up to *E*_F_ (gray filling). The pump pulse perturbs the probe absorption by promoting e from VB to CB (red filling), which then relax through emission of optical phonons (downward black arrows). The three sketches correspond to (a) *E*_F_ at the Dirac point, (b,c) moderate *n-*doping with *E*_F_ (b) below and (c) above the threshold for interband probe absorption. By increasing *E*_F_, optical phonon emission is quenched (dashed downward arrows) and relaxation becomes slower. Above the threshold for interband absorption of the probe (dashed vertical blue arrow), photoexcitation results in Δ*T*/*T* < 0, leading to reverse saturable absorption, consisting in an increased absorption upon increasing illumination.

The electrical tunability of the SLG relaxation dynamics, sketched in Figure 6a–c, is promising for the realization of tunable SA. Saturable absorption, i.e., the quenching of optical absorbance under intense illumination,^101^ can occur in SLG at low light intensity (e.g., ∼ 0.750MW cm^–2^ at 0.8 eV^102^). We measured a saturation intensity^44^*I*_S_ = 0.5–1.7MW cm^–2^ for photon energies in the range ∼0.5–2.5 eV, comparable to semiconductor saturable absorber mirrors (SESAMs) (*P* = 0.01–0.1MW cm^–2^ at 0.944 eV^103^), but maintained over a much broader spectral range.^44^ The modulation depth, defined as the maximum change in absorption,^101^ can be optically tuned exploiting cross absorption modulation.^104^ GSAs are promising for passive mode-locking,^44,105,106^ Q-switching,^107^ and Q-switched mode-locking.^108^

Figure 6 shows that the SLG equilibrium photoresponse can be electrically tuned, providing an additional knob for controlling its SA performance in terms of modulation depth and recovery dynamics. For *E*_F_ ≪ *ℏω*_probe_/2, the intrinsic biexponential-like relaxation dynamics makes SLG an ideal fast SA, Figure 6a. The presence of two different time scales, in analogy with SESAMs,^109^ is considered an advantage for mode locking.^109^ As discussed in refs (109) and (110), the longer time scale reduces the saturation intensity, facilitating self-starting mode-locking, while the fast relaxation component is efficient in shaping subps pulses. For *E*_F_ ≤ *ℏω*_probe_/2 as in Figure 6b, SLG can act as slow SA^111^ with recovery times 10 to 30 times longer than the pulse duration,^111,112^ favoring soliton shaping,^112^ or the temporal shift of the pulses caused by the SA,^109^ which limits the time in which noise behind the pulse can be amplified.^111^ Longer recovery time also gives an increased tolerance towards instability induced by self-phase modulation.^111^

The PA at *E*_F_ > *ℏω*_probe_/2 can be exploited to operate SLG as reverse SA,^113^ for which absorption increases with increasing impinging intensity, because of depletion of the final state population (see Figure 6c). The PA of highly doped SLG could be exploited to realize an optical limiter,^114^ based on the decrease in transmittance under high-intensity or fluence illumination. An ideal optical limiter, with the functionality of protecting delicate optical elements, should strongly attenuate intense, potentially dangerous, laser beams, while exhibiting high transmittance for low-intensity light. Carbon nanotubes^115^ and few-layer graphene^116^ dispersions in organic solvents have been used to prepare optical limiters. However, these rely on nonlinear scattering,^117^ rather than on nonlinear absorption.^118^ The nonlinear scattering of graphene dispersions^116^ is based on the avalanche ionization of carbon when interacting with an incident laser pulse, and subsequent bubble formation in the solvent due to the heat released by expanding microplasmas.^115,116^ The 10 ps PA lifetime of the nonlinear absorption of highly doped SLG is 10 times shorter than the typical time scales for thermal effects and bubbling of graphene dispersions, which are of the order of 100 ps,^115^ allowing the application to lasers with shorter pulse duration. The nonlinear absorption in SLG is not related to a specific absorption resonance. Thus, it covers a broad spectral range, as shown in Figure 3b, where for |*E*_F_| = 600 meV we detect PA for photon energies in the range 0.729 to 1.127 eV. The SA to reverse SA transition could be used for all-optical logic gates.^119^ Gate-dependent effects on cooling dynamics are also important for the design of transceivers for data communication.^3^ E.g., ref (120) showed that longer cooling times give larger photocurrent.

## Conclusions

We demonstrated that electrostatic tuning of the nonequilibrium optical response of SLG results in changes of amplitude, sign, and recovery dynamics of Δ*T*/*T*. Increasing *E*_F_ quenches emission of optical phonons, i.e., of the fastest intrinsic relaxation channel for SLG hot charge carriers. The ability to tune *E*_F_ above the threshold for Pauli blocking of interband absorption of NIR light results in photoinduced absorption in SLG, because of pump-induced unblocking of interband transitions for the probe. Our results anticipate the use of voltage-controlled SLG for nonequilibrium optoelectronic devices as gate tunable optical elements, which can behave either as fast, slow, or reverse SA.

## Methods

### High-Sensitivity Transient Absorption Microscopy

The setup for pump–probe experiments comprises a mode-locked Er-doped fiber oscillator (Toptica Photonics, FemtoFiberPro), emitting 150 fs pulses at 0.8 eV (1550 nm) at 40 MHz repetition rate. The oscillator feeds two Er-doped fiber amplifiers (EDFAs) each generating 70 fs pulses at 0.8 eV with 300 mW average power. The output of the first EDFA is attenuated to obtain pump pulses with 1 mW maximum average power. The second EDFA feeds a highly nonlinear optical fiber that produces a supercontinuum tunable between 0.729 and 1.240 eV, which serves as probe pulse. The pump and probe pulses, synchronized by a computer-controlled optical delay line and collinearly recombined by a dichroic beam splitter, are focused on the sample over spots of ∼25 μm radius. The portion of the probe transmitted by the sample, spectrally selected by a monochromator with a bandwidth ∼5 nm, is detected by an amplified InGaAs photodiode (bandpass 4.5 MHz, gain 10^4^) and analyzed by a lock-in amplifier (Zurich Instruments HF). Pump and probe pulses have perpendicular polarizations and a linear polarizer is used to filter out the pump light scattered from the sample. The pump pulse is modulated at 1 MHz by an acousto-optic modulator, resulting in a Δ*T*(*t*)/*T* sensitivity of the order of 10^–7^, for an integration time of 300 ms. From the FWHM of the instrumental response function, we estimate an overall temporal resolution ∼100 fs. The absorbed photon density is in the range 2–3 × 10^12^cm^–2^ (depending on *E*_F_), as calculated from incident fluence and sample transmission.

### Simulation of Differential Transmission Dynamics

To model the time-evolution of the differential transmission we assume that, on the time-scale given by the time-resolution of the experiment (100 fs), e in both CB and VB are thermalized at the same *T*_e_, and reach a common μ_*c*_(*t*), such that the e energy distribution is a HFD. μ_*c*_(*t*) is calculated at each instant in time, as it depends on *T*_e_ and is fixed by the condition that the carrier density, defined as^94^, with ν(ε) the electronic density of states in SLG,^122^ is constant.^94^ As for refs (98) and (121), we can write the following EOMs for *T*_e_ and phonon occupations:
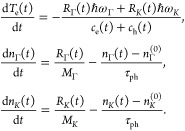
1Here, *n*_Γ_(*t*) and *n*_*K*_(*t*) are the occupations of the optical phonon modes at Γ and *K*, with energy *ℏω*_Γ_ ∼ 0.196 eV and *ℏω*_*K*_ ∼ 0.161 eV,^100^ respectively, as these have the strongest electron–phonon coupling.^100^ The parameter τ_ph_ is the finite optical phonon lifetime, via relaxation into acoustic phonons due nonlinearities of the lattice,^65^ until global thermal equilibrium with densities *n*_Γ_^(0)^, *n*_*K*_^(0)^ is reached. We find good agreement between theory and experiment for τ_ph_ ∼ 1.2 ps, consistent with ref (65). The constant coefficients *M*_Γ_, *M*_*K*_ correspond to the number of phonon modes in an annular region between the minimum and maximum energy that can be exchanged with e.^98,121^ The time-dependent parameters *c*_e_(*t*) and *c*_h_(*t*) are the heat capacities of e in CB and h in VB, respectively. The time-dependent parameters *R*_Γ_(*t*) and *R*_*K*_(*t*) are electronic relaxation rates per unit area, due to phonon emission and absorption, proportional to a Boltzmann scattering integral.^98,121^

In line with our assumption that, on the time-scale probed by our experiments, a common μ_*c*_(*t*) is established between CB and VB, the heat capacities are calculated separately in the two bands, (i.e., *T*_e_ variations are decoupled from interband transitions) and only intraband transitions are included in the relaxation rates. The initial *T*_e_(0), following the pump pulse, is estimated as for ref (37). The initial phonon populations *n*_Γ,*K*_(0) are evaluated at RT. The optical photoconductivity Δσ(*t*) = σ(*t*) – σ(0)^122^ depends on *T*_e_(*t*) and μ_*c*_(*t*). We use the Tinkham formula^123^ to obtain the differential transmission.
